# Hydroclimatic and cultural instability in northeastern North America during the last millennium

**DOI:** 10.1371/journal.pone.0248060

**Published:** 2021-03-26

**Authors:** J. Curt Stager, Brendan Wiltse, Brian F. Cumming, Timothy C. Messner, Joshua Robtoy, Sidney Cushing

**Affiliations:** 1 Natural Sciences, Paul Smith’s College, Paul Smiths, NY, United States of America; 2 Department of Biology, Queen’s University, Kingston, ON, Canada; 3 Department of Anthropology, SUNY Potsdam, Potsdam, NY, United States of America; Woods Hole Oceanographic Institution, UNITED STATES

## Abstract

Long-term, large-scale perspectives are necessary for understanding climate variability and its effects on ecosystems and cultures. Tree ring records of the Medieval Climate Anomaly (MCA) and Little Ice Age (LIA) have documented major hydroclimatic variability during the last millennium in the American West, but fewer continuous, high-resolution hydroclimate records of the MCA-LIA period are available for eastern North America, particularly during the transition from the MCA to the LIA (ca. A.D. 1250–1400). Diatoms (micro-algae with silica cell walls) in sediment cores from three Adirondack (NY, USA) lakes and a hiatus in a wetland peat deposit in the Adirondack uplands provide novel insights into the late Holocene hydroclimate history of the Northeast. These records demonstrate that two of the region’s most extreme decadal-scale droughts of the last millennium occurred ca. A.D. 1260–1330 and ca. A.D. 1360–1390 during a dry-wet-dry (DWD) oscillation in the Adirondacks that contributed to forest fires and desiccation of wetlands in New York and Maine. The bimodal drying was probably related to more extreme droughts farther west and coincided with major events in Iroquoian and Abenaki cultural history. Although the causes of the DWD oscillation in the Adirondacks remain uncertain, changing sea-surface temperatures and solar variability are likely to have played a role.

## Introduction

Paleoclimate reconstructions of the last millennium indicate generally warmer conditions in much of North America during the Medieval Climate Anomaly (MCA), which is commonly defined as the period between A.D. 950 and A.D. 1250 [1], followed by cooler conditions during the Little Ice Age (LIA) ca. A.D. 1400–1700 [1–3]. Precipitation, however, is less regionally coherent than temperature and therefore requires denser networks of proxy records to generate reliable reconstructions [4]. Although reconstructions of regional moisture balance from the Northeast, defined here as the eastern Great Lakes region through New England, are available from upstate New York [5–8], southern Ontario [9] and New England [10–17], they are much less numerous than in the American West where more long-lived, drought-sensitive trees are available for dendroclimatic analysis [4, 17], and many of the records are discontinuous, relatively short, or lacking in fine-scale temporal resolution [17]. In addition, the Northeast receives more abundant precipitation year-round from many sources including the Pacific, Gulf of Mexico, Great Lakes, and North Atlantic, making causal attributions of drought or unusually wet conditions more difficult [5, 12, 16, 17]. As a result, the nature, causes, and consequences of late Holocene hydroclimatic variability in the Northeast—and therefore the interconnected climate systems of North America as a whole—remain to be fully characterized, particularly during the transitional period between the MCA and LIA which is studied less frequently than the MCA and LIA proper.

Here we use diatom assemblages in sediment cores from three Adirondack lakes to reconstruct relative changes in hydroclimatic conditions through the MCA-LIA transition with decadal to sub-decadal resolution. The ratios of planktonic to benthic taxa (%P) in diatom assemblages are highly sensitive to light penetration of the water column that drives algal photosynthesis in habitats across a range of depths [5, 11, 18–20]. Reduced water clarity or deepening therefore tend to be associated with decreased relative abundances of benthic taxa (higher %P) at coring sites as is lake expansion, which can shift littoral habitats outward from lake margins and reduce hydrodynamic transport of benthic diatoms to deposition centers offshore [5, 11, 18–20]. Such changes typically result from periods of increased regional moisture balance that, in addition to increasing water volume, can also hinder light penetration by increasing inputs of algae-stimulating nutrients and pigmented soil carbon compounds [20–22]. Percentages of planktonic diatoms in sediment assemblages are therefore potentially useful for tracking hydroclimate variability over long time periods [5, 18, 19, 22, 23].

Our diatom-based inferences are strictly qualitative due to multiple potential influences on light penetration other than total precipitation and water depth alone, including darkening by dissolved organic carbon, phytoplankton productivity, and siltation under wetter, stormier conditions [19, 24, 25]. Nonetheless, regionally coherent changes in the diatom records of hydrologically distinct lakes can be used in combination with one another to identify relative shifts in hydroclimate regimes, particularly decadal-scale droughts that have previously been overlooked in many records from northeastern North America.

In this study we use variability in a high-resolution planktonic diatom record from Little Green Pond, NY, supported by diatom records from two additional lakes and a wetland from the Adirondack uplands, to show that pronounced aridity occurred across much of the Northeast during the MCA-LIA transition. In particular, two dry periods ca. A.D. 1260–1330 and ca. A.D. 1360–1390 represented some of the region’s most severe multi-decadal droughts of the last millennium. We also demonstrate that the dry-wet-dry (DWD) oscillation was temporally linked to more extreme "megadroughts" [17, 26] farther west, potentially influenced by multiple climatic forcing mechanisms, and contemporaneous with significant events in North American cultural prehistory.

### Study sites

**Fig 1:**
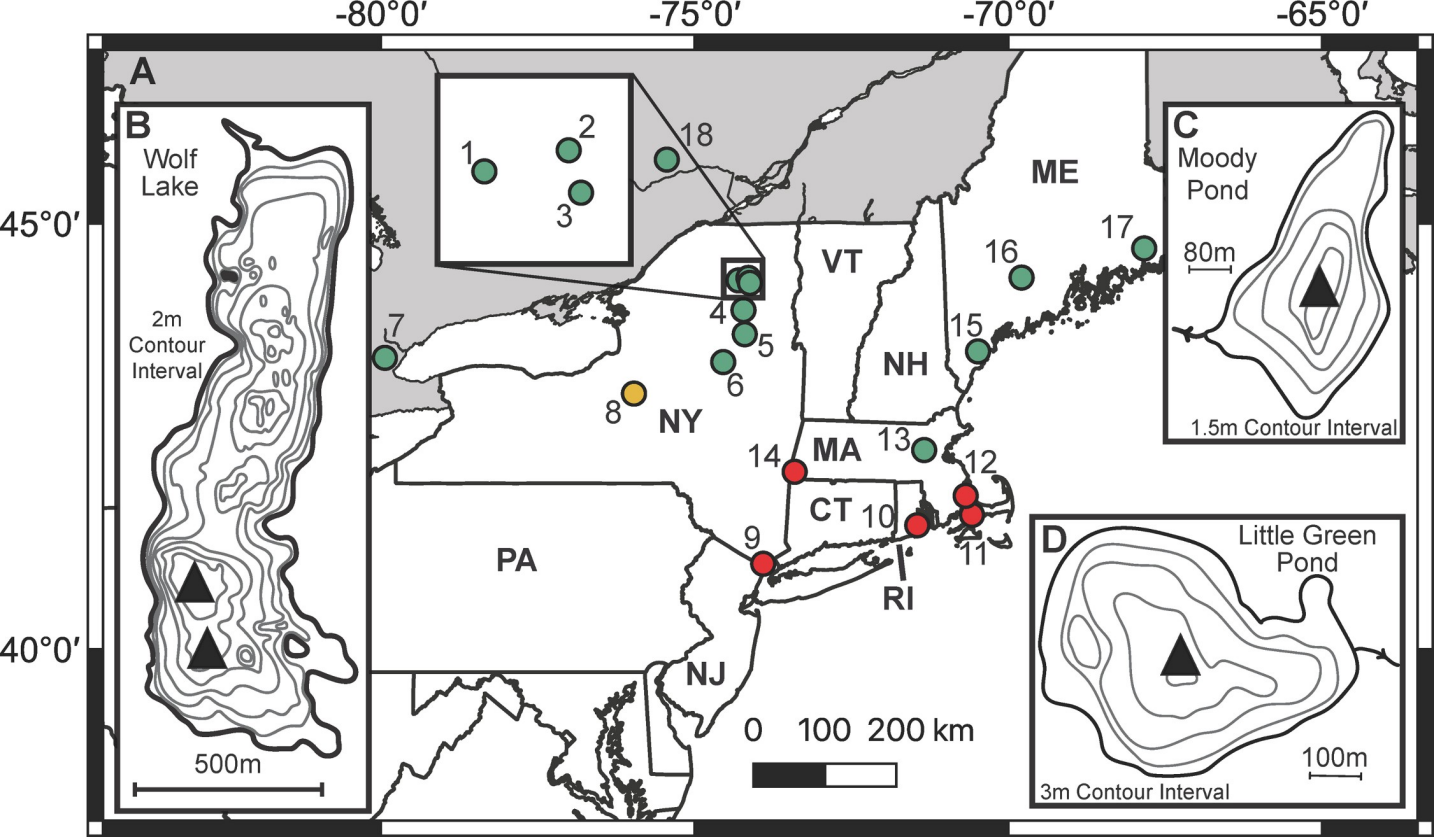
Site maps. Panel A. Northeastern region with locations of paleoclimate study sites referred to in the text. Green sites registered one or both of the dry phases of the DWD oscillation, red sites did not, and the yellow site was equivocal. Panels B, C, D. Contour maps of Wolf Lake, Moody Pond, and Little Green Pond respectively, with coring sites indicated by black triangles. Study sites shown on regional map: 1. Little Green Pond, 2. Bloomingdale Bog, 3. Moody Pond, 4. Wolf Lake, 5. Clear Pond [8], 6. Piseco Lake [7], 7. Crawford Lake, marking the eastern margin of the study area used by Buckley et al. [9], 8. Fayetteville Green Lake [12], 9. Piermont Marsh [3], 10. Pettaquamscutt River Estuary [12], 11. Deep Pond [13], 12. New Long Pond [14], 13. Walden Pond [11], 14. Davis Pond [14], 15–17. Saco Heath, Sidney Bog, Great Heath Bog, respectively [10]. 18. Lac Brulé [37].

Little Green Pond and the two supporting lakes were selected on the basis of shared limnological traits including relatively high transparency (Secchi disk depths in the 4–8 m range), modest size and depth, a small surface outlet, relatively simple bathymetric contours, lack of major tributaries, circumneutral pH, and similar planktonic diatom communities. All three sites are located within the central uplands of the 2.5 million ha Adirondack Park (Fig 1) where bedrock is primarily granitic and therefore free of carbonate minerals containing ancient carbon that can complicate radiocarbon dating of lake sediments.

The four sites selected for this study are located within less than 40 km of one another in the Adirondack uplands (Fig 1). All are hydrologically distinct from one another but their climatic settings are very similar [5, 27]. Mean annual temperature in the uplands is ca. 4°C, and mean annual precipitation is ca. 100 cm/yr [27]. Prevailing storm tracks are westerly, but precipitation can come from multiple sources including the Gulf of Mexico, tropical Atlantic, and North Atlantic as well as the Pacific.

Little Green Pond (44°21’26"N, 74°17’58"W) has a surface area of 28 ha and maximum depth of 12.5 m, and is situated in a glacial kettle depression at 488 meters above mean sea level (m.a.m.s.l.) within a forested watershed (Fig 1). The pond has been subjected to rotenone treatments and stocking with various fish species since the mid-20^th^ century. Moody Pond (44°19’43"N, 74°07’06"W) is a 9 ha lake with a maximum depth of 5 m, situated at 470 m.a.m.s.l. within a largely forested watershed (Fig 1). It has experienced significant human impacts including shoreline development, moderate eutrophication, invasive species, and fish stocking during the last century. Wolf Lake (44°01’03"N, 74°13’14"W) is a remote lake of 62 ha in a forested watershed at 556 m.a.m.s.l. (Fig 1). Because access to the lake is strictly controlled by the New York State University of Environmental Science and Forestry, it is an exceptionally undisturbed "heritage lake" [5, 28] that has been protected from fisheries management practices, shoreline development, damming, and other local-scale human impacts. Correlations between %P in surface sediment samples and water depth at Wolf Lake were very strong (r^2^ = 0.96) when sampled in 2012, and %P in sediment core assemblages also tracked local precipitation variability during the mid- to late 20^th^ century [5]. Bloomingdale Bog (44°23’03"N, 74°08’25"W) is a raised wetland of ca. 100 ha at ca. 480 m a.m.s.l. that empties into the Saranac River drainage (Fig 1). Vegetation includes *Sphagnum*, grasses, and ericaceous shrubs, and depths of the peat deposits generally range from 1 to 2.5 m.

## Materials and methods

Little Green Pond core LG-P (105 cm long) was collected in 2014 from 12 m depth in the center of the pond (Fig 1) with a modified Kullenberg sampler and extruded vertically in the field in 0.25 cm increments. Supporting core LG-2 was collected from the same location in 2012 with a Glew gravity sampler. Supporting core WOLF-17B (77 cm long) was collected from 12.5 m depth in the central basin of Wolf Lake in 2017 with a modified Kullenberg sampler, and supporting core MOODY-C (45 cm) was collected in 2019 from 5 m depth in the center of Moody Pond with a Glew gravity sampler. All three supporting cores were extruded vertically in the field in 1 cm increments. The peat core from Bloomingdale Bog (50 cm) was collected from the approximate center of the wetland with a Russian peat sampler after hand-removal of 35 cm of loose *Sphagnum* that lay atop denser peat below (total section length = 85 cm). Collection and possession permit #1313 was provided to Paul Smith’s College by the NYS Department of Environmental Conservation for field sampling.

A mean of 1000 diatom valves per sample from the LG-P core were identified at 1000X under oil immersion using standard references [29–32]. Diatom assemblages were enumerated at 0.25 cm intervals between 15 and 65 cm depth and at coarser resolution (0.5–1.0 cm) above and below that. Siliceous scales of chrysophyte algae were also enumerated along with the diatoms. Planktonic diatom assemblages in cores LG-2, WOLF-17B, and MOODY-C were examined at coarser temporal resolution for comparative purposes only, with a mean of 300 diatom valves counted per sample.

### Age control

**Fig 2:**
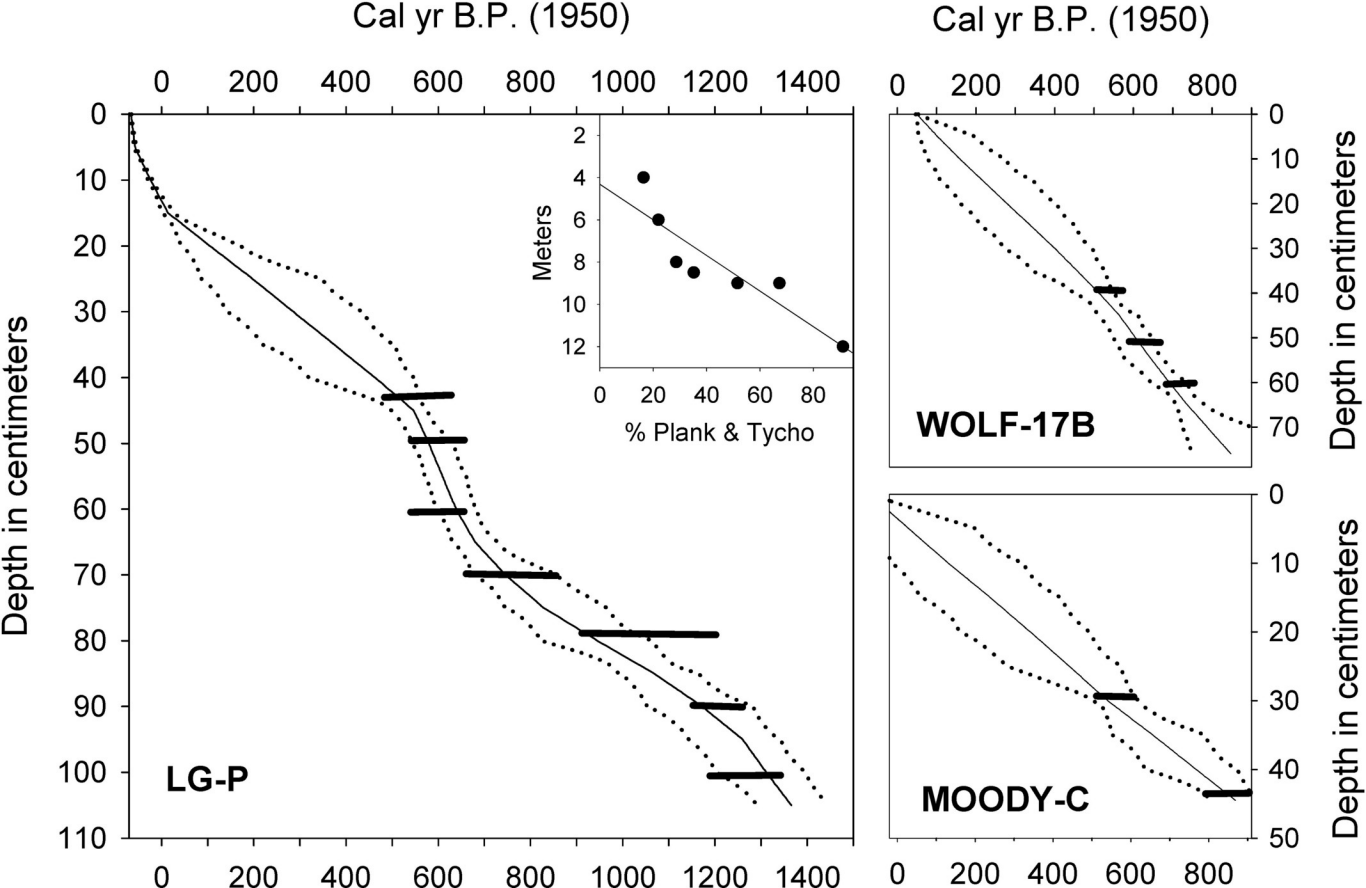
Radiocarbon age-depth models for sediment cores from Little Green Pond (LG-P), Wolf Lake (WOLF-17B), and Moody Pond (MOODY-C). Horizontal bars represent calibrated age probability ranges (2-σ) for the radiometric dates. Dotted (solid) lines represent maximum-minimum (mean) ages. Inset: percentages of planktonic and tychoplanktonic diatoms in surface sediment assemblages along a water depth gradient in Little Green Pond.

**Fig 3:**
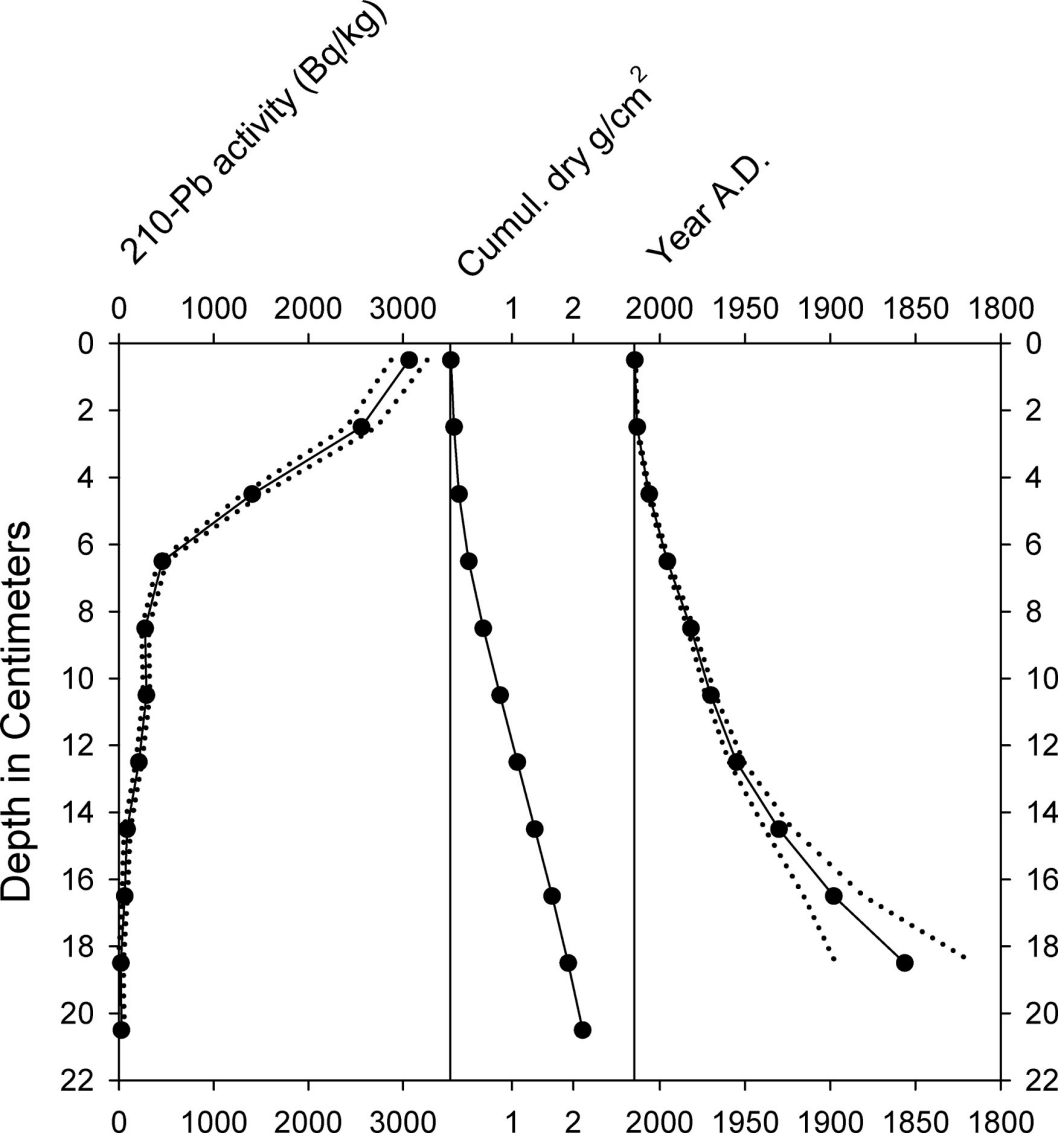
Activity of ^210^Pb, sediment accumulation rates, and age-depth relationships in Little Green Pond core LG-2. Dotted lines indicate maximum/minimum ranges of the ^210^Pb activities and radiometric dates.

Bayesian age-modeling of the LG-P core (Fig 2) was conducted with the BACON version 2.3.9.1 modeling package [33]. The age-depth model for LG-P was based upon accelerator mass spectrometry (AMS) ages of seven bulk sediment samples (Table 1) and comparison of the recent diatom stratigraphy with that of gravity core LG-2. The gravity core was dated with ^210^Pb (Fig 3) using a germanium well detector for gamma counting at Queen’s University and a constant rate of supply model was used to establish an age-depth chronology [34]. Because stratigraphic comparison of diatom records from the two cores indicated that the mud-water interface of LG-P was intact, the age of the top of that core was taken to be 2014, the year of collection.

**Table 1:** Radiocarbon ages of Adirondack core subsamples.

Sample (cm)	delta-^13^C (o/oo)	^14^C y B.P.	Cal y A.D. range (probability)	Lab ID
*LG-P*				
42.5–43.5	-28.8	500 ± 30	1334–1336 (0.01)	Beta-414280
			1398–1448 (0.99)	
49.5–50.5	-28.5	590 ± 30	1299–1370 (0.71)	Beta-414281
			1379–1413 (0.29)	
59.5–60.5	-28.5	590 ± 30	1299–1370 (0.71)	Beta-404202
			1379–1413 (0.29)	
69.5–70.5	-28.9	840 ± 30	1059–1063 (0.01)	Beta-414282
			1154–1264 (0.99)	
79.5–80.5	-28.4	1080 ± 30	894–930 (0.28)	Beta-414283
			937–1018 (0.72)	
89.5–90.5	-28.7	1280 ± 30	664–773 (1.00)	Beta-414284
99.5–100.5	-27.5	1360 ± 30	616–693 (0.97)	Beta-404203
			748–762 (0.03)	
*Moody-C*				
30–31	na	535 ± 15	1331–1339 (0.04)	OS-154248
			1397–1430 (0.96)	
43–44	na	935 ± 15	1035–1059 (0.20)	OS-151751
			1064–1154 (0.80)	
*Wolf-17B*				
40–41	na	475 ± 25	1414–1443 (1.00)	OS-141068
51–52	na	575 ± 15	1316–1354 (0.64)	OS-142607
			1389–1411 (0.36)	
60–61	na	765 ± 15	1224–1234 (0.06)	OS-141069
			1242–1278 (0.94)	
*Bloomingdale*				
56.5–57.5	-27.0	730 ± 30	1224–1237 (0.03)	Beta-315543
			1241–1297 (0.97)	
58.5–59.5	-28.9	2200 ± 30	B.C. 366–191 (0.99)	Beta-315542
			B.C. 188–186 (0.01)	

Calibrated 2σ age probability ranges determined with CALIB 7.1.0 [35]. All of the samples analyzed from Little Green Pond and Moody Pond were bulk organic lake sediment. The samples analyzed from WOLF-17B were pollen fractions, and the Bloomingdale Bog samples consisted of fibrous peat (i.e. plant macrofossils).

Bayesian age modeling of supporting core Moody-C was based on AMS dates from two bulk sediment samples (Table 1) and similar age-modeling for WOLF-17B was based on AMS ages of three pollen samples (Table 1). Comparison with the diatom stratigraphy of WOLF-62 [5] and other (unpublished) cores from this lake suggested that the last ca. 100 years of the diatom record (ca. 10 cm) of sediment were missing from the top of WOLF-17B. Therefore, an approximate date of A.D. 1900 was estimated for the core top. Fibrous peat samples from 1 cm above and below a textural transition upwards from relatively woody to more fibrous herbaceous peat at 62 cm depth in the Bloomingdale Bog core were collected for radiometric dating of plant remains (Table 1).

## Results

In general, the radiocarbon-based age-depth profiles of LG-P, MOODY-C, and WOLF-17B revealed relatively smoothly increasing ages with depth (Fig 2). However, the LG-P profile displayed more variability than the other two, which could reflect changing sedimentation regimes and/or slight offsets due to reworking of formerly deposited organic matter within the lake. The basal age of core LG-P was ca. 1360 calibrated years before present (BP), and the extrapolated basal ages of supporting cores MOODY-C and WOLF-17B were ca. 1100 and 1050 cal yr BP, respectively.

Radiocarbon ages of peat immediately above and below the textural transition at 58 cm depth in the Bloomingdale Bog core indicated that roughly 1500 years were missing from the record and that peat deposition resumed during the mid- to late 13^th^ century (Table 1). A similar hiatus due to fire and/or drought was also encountered in peat cores taken independently from elsewhere in the bog [6].

The activity of ^210^Pb in Little Green Pond core LG-2 decreased exponentially with depth, reaching background levels within the upper ca. 20 cm of the core (Fig 3). The base of the core was deposited during the early to mid-18^th^ century.

### Diatom assemblages

**Fig 4:**
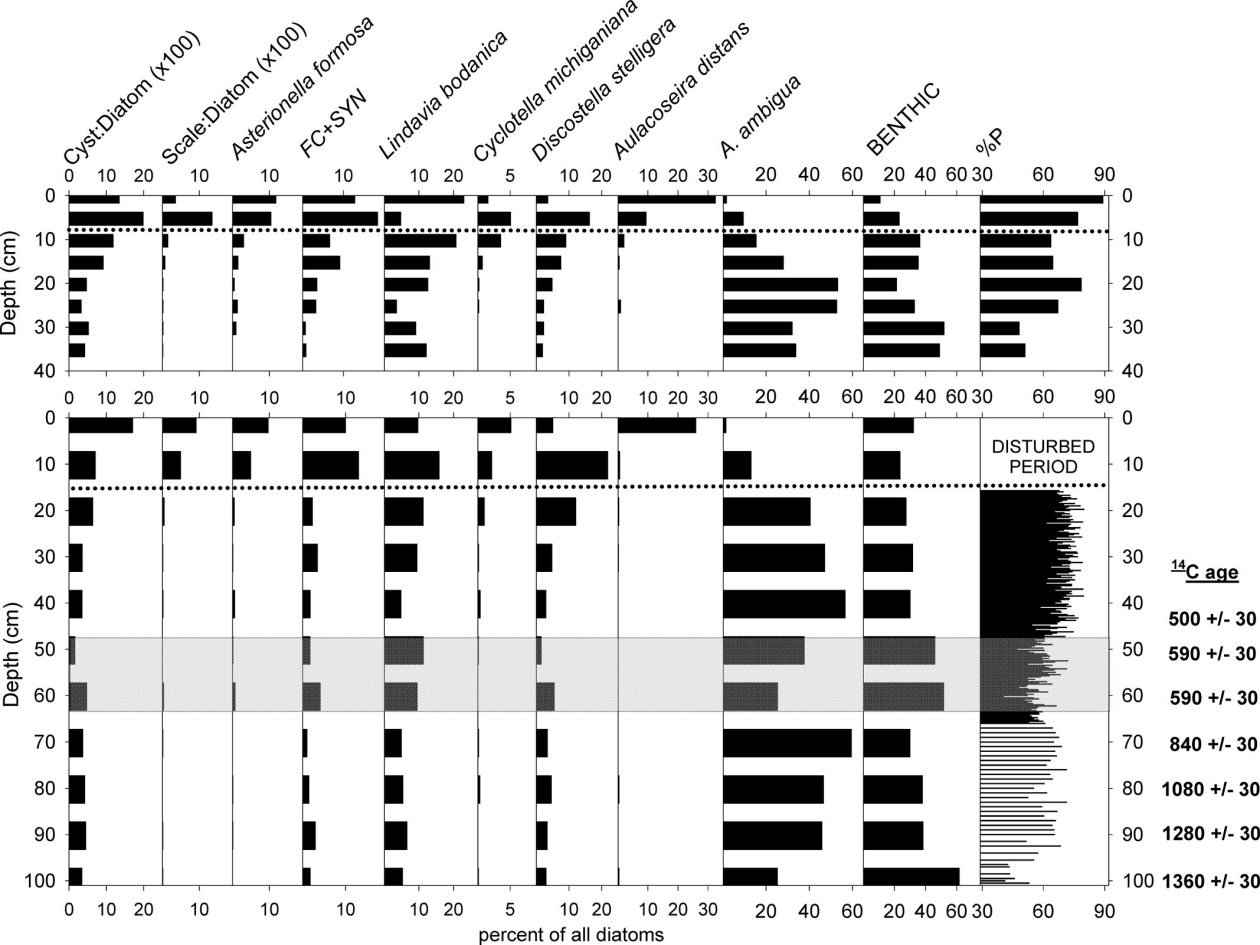
Microfossil assemblages in Little Green Pond cores LG-2 (top) and LG-P (bottom). Cyst:Diatom, Scale:Diatom = ratios of chrysophyte algal cysts and scales to diatoms. All diatom taxa are planktonic or facultatively planktonic except for the "BENTHIC" category. FC+SYN = sum of *Fragilaria crotonensis* and *Synedra* spp. percentages. Dotted line designates disruption of diatom communities by fisheries management practices during the A.D. 1950s. The presence of similar anthropogenic disturbance intervals in both cores demonstrates that the top of LG-P was intact. Horizontal grey bar highlights the DWD interval discussed in the text. Radiocarbon ages for LG-P are provided adjacent to their corresponding depths in the figure.

In Little Green Pond cores LG-2 and LG-P, the %P fractions of the diatom assemblages were primarily composed of *Aulacoseira ambigua*, *Discostella stelligera*, and the *Lindavia bodanica* group (Fig 4). However, members of the facultatively planktonic *A*. *distans* group along with planktonic *Asterionella formosa*, *Fragilaria crotonensis*, and long *Synedra* spp. cf. *S*. *nana*) also became more abundant in the upper 15 cm of the cores. The latter three planktonic taxa are commonly found in lakes that have undergone cultural eutrophication or other anthropogenic disturbances [11, 36], and their increased prevalence in the diatom assemblages probably reflects disruption of the pond ecosystem by fisheries management practices since the 1950s. Because such changes would have obscured climate-related shifts in the diatom assemblages, we did not evaluate the most recent portion of these records in terms of hydroclimate. Analysis of diatoms of sediment samples from the mud-water interface along a depth transect in Little Green Pond yielded strong correlations of %P with water depth (Fig 2; r^2^ = 0.82) similar to those found previously in Wolf Lake [5].

**Fig 5:**
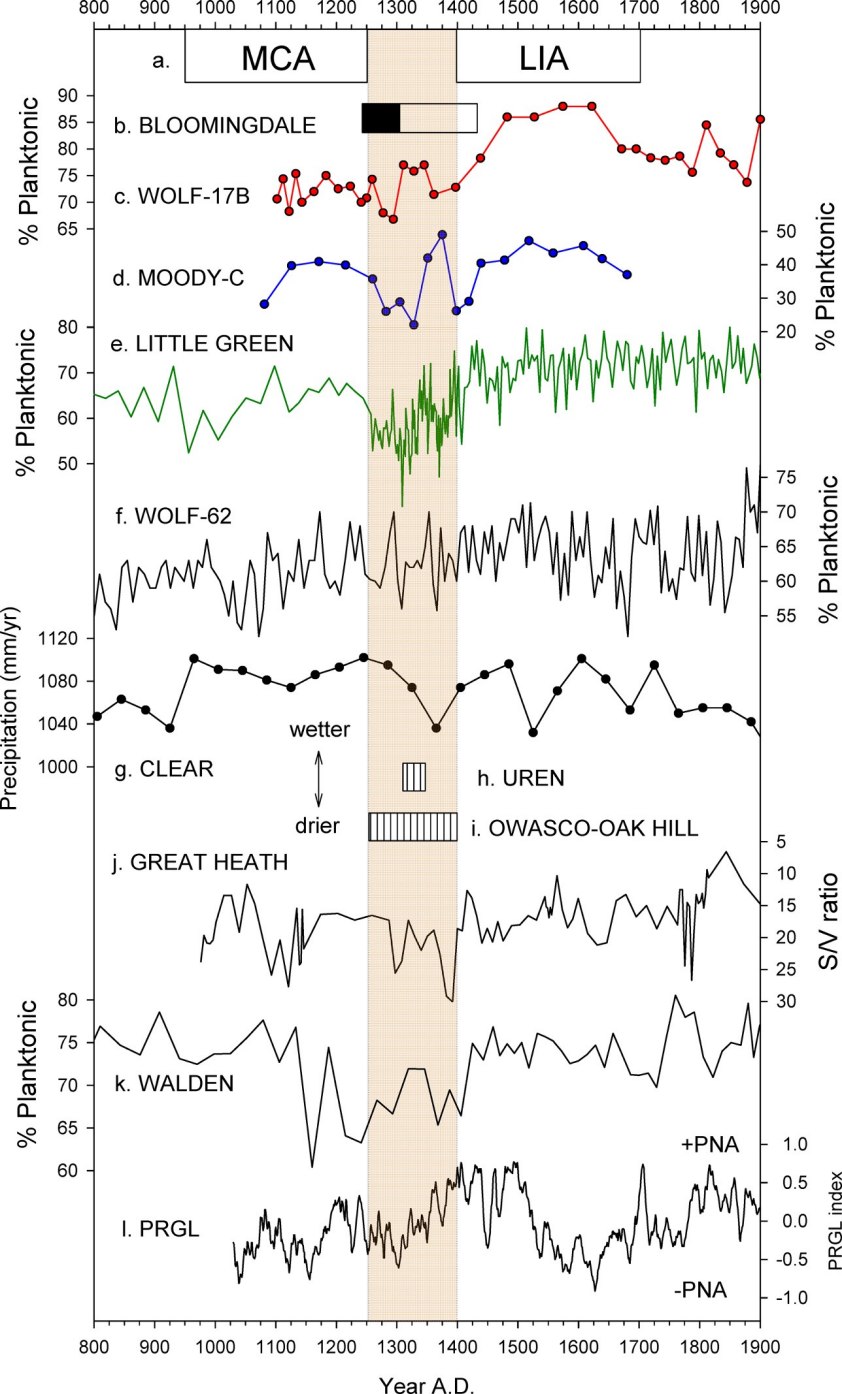
Paleoclimatic and cultural records of the last millennium. (a) MCA = Medieval Climate Anomaly, LIA = Little Ice Age. Tan column spans the MCA-LIA transition A.D. 1250–1400. (b) Age probability ranges for the resumption of peat deposition in Bloomingdale Bog after hiatus. Black box = this study, open box = [6]. (c-e) %P in cores from Wolf Lake, Moody Pond, and Little Green Pond, respectively (this paper). Chronologies are based on mean ages with multidecadal uncertainty ranges that could allow closer temporal alignment of peaks. (f) %P in core WOLF-62 [5]. (g) Pollen-derived precipitation record from Clear Pond, NY [8]. (h, i) Cultural periods among Iroquoian cultures in southern Ontario [59]. (j) Hydrological reconstruction from Great Heath Bog, ME [10]. (k) %P in core from Walden Pond, MA [11]. (l) Composite PNA pattern reconstruction from NY and RI [12]. All proxy records are arranged with wetter conditions upward.

The most common planktonic diatoms in core MOODY-C were *Tabellaria flocculosa* var. IIIP, *Aulacoseira ambigua*, *A*. *distans* complex, *Discostella stelligera*, and the *Lindavia bodanica* group. In Wolf Lake the dominant planktonic taxa were *D*. *stelligera*, *L*. *bodanica*, *T*. *flocculosa*, *A*. *ambigua*, *A*. *subarctica*, and *A*. *lirata* (Fig 5) but %P was higher in core WOLF-17B than WOLF-62 [5] due to its deeper setting.

For this study, we focus primarily on the 66–45 cm (ca. A.D. 1250–1400) interval in core LG-P within which large decreases in %P occurred. Mean %P fell from 66% overall to 56% (41–66%) between 66 and 56 cm (ca. A.D. 1260–1330), the lowest values of the last millennium. Between 52 and 47 cm (ca. A.D. 1360–1390) %P declined to a mean of 58% (47–64%), and values averaged 63% (52–72%) within the intervening interval (Fig 5).

Similar bimodal decreases in %P occurred in the less finely resolved diatom records of Wolf Lake and Moody Pond (Fig 5). In all three lake records, the first decrease in %P was more extreme than the second and represented the lowest values of the last millennium. A >1400 yr hiatus in the Bloomingdale Bog core (Table 1) indicated resumption of peat deposition, most likely after fire and/or drought, during the mid- to late 13^th^ century (Fig 5).

## Discussion

Radiocarbon-based age models yield somewhat "floating" chronologies due to various error sources including reworking of organic compounds, sediments, or botanical remains as well as the inherent uncertainty of radiometric dating illustrated by the multidecadal age ranges in our records (Table 1). Although these dates are less firmly anchored in time than those from most tree rings records, we are confident that ancient carbon offsets are not significant in Wolf Lake because equivalent dates were previously obtained for pollen and bulk sediment samples there [5], and the similar ages and durations of similar excursions in %P at all three lakes in addition to the hiatus at Bloomingdale Bog strongly suggest that the excursions were contemporaneous. We therefore conclude that these multi-proxy signals of drought from multiple locations are best explained by synchronous regional responses to a dry-wet-dry climatic oscillation during the MCA-LIA transition.

Few high-resolution paleoclimate reconstructions of the last millennium are available from the Adirondack region for comparison to our hydroclimate records. The diatom record of WOLF-17B revealed the DWD oscillation more clearly than that of core WOLF-62 (Fig 5), which was collected from a shallower portion of the lake where fluctuations in %P were more muted [5]. Resumption of peat deposition above a hiatus at similar depth to ours in another core from Bloomingdale Bog [6] was said to occur ca. A.D. 1400, but the calibrated probability distribution of the radiocarbon date upon which that age was based spanned A.D. 1299–1435, which overlapped with the DWD oscillation (Fig 5). Two prominent charcoal deposition peaks in a core from Piseco Lake, NY, indicated forest fires ca. A.D. 1300 and 1400 that appear to have coincided with the bimodal droughts [7]. A pollen-based record from Clear Pond, N.Y. [8] registered a single broad precipitation decrease during the 14^th^ century (Fig 5), and fluctuations in varve thickness and composition at Fayetteville Green Lake, NY [12], were also consistent with evidence for a DWD oscillation in the Adirondacks. However, a composite "PRGL" series derived from the Green Lake data in combination with a record from coastal Rhode Island [12] exhibited a prominent drought signal only during the first half of the DWD interval (Fig 5). A pollen record from the Hudson River estuary (Fig 1) that was taken to indicate increased moisture during the 14^th^ century displayed no clear evidence of a DWD oscillation [3].

Elsewhere in the Northeast, a similar DWD oscillation was registered at Walden Pond, MA, (Figs 1 and 5) [11] and severe drying in Maine ca. A.D. 1300 and A.D. 1400 contributed to forest fires and drought-related hiatuses in peatland deposits similar to those in the Adirondacks (Figs 1 and 5) [10]. Tree-ring series from southern Ontario [9] indicated moderate drying A.D. 1280–1320 and A.D. 1387–1396, but only two of the seven chronologies covered the DWD interval and they differed in the relative intensities of the dry phases. A varved pollen record from Lac Brulë, southern Quebec, registered a single dry period ca. AD 1400 [37]. Most other northeastern paleoclimate records of the last millennium lack the temporal resolution or hydroclimate-sensitivity needed to detect the DWD, but sedimentological time series from Davis Pond, Deep Pond, and New Long Pond, MA (Fig 1), registered no major low stands during the DWD interval [13, 14]. These discrepancies among lacustrine records might reflect true geographic variability in hydroclimate, because most of the sites lacking the DWD signal were restricted to the southern sector of the region from Cape Cod and Rhode Island to the Hudson estuary (Fig 1). Alternatively, they might reflect differing sensitivities of proxies and/or coring sites as was the case with the two diatom records from Wolf Lake (Fig 5).

### Continental patterns

**Fig 6:**
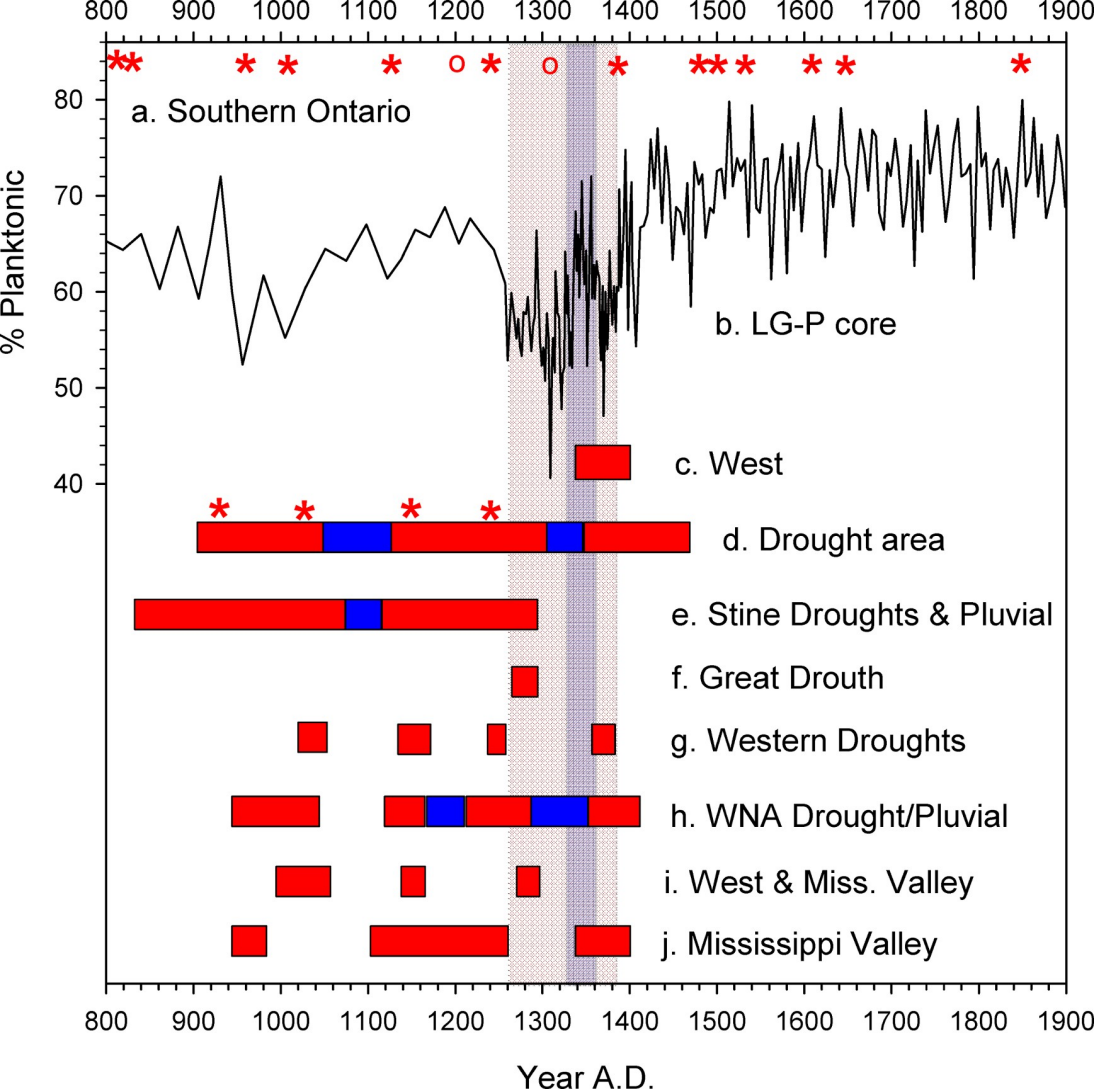
Hydroclimate variability at Little Green Pond in comparison to tree ring-based hydroclimate reconstructions from western North America and the Mississippi Valley. Vertical bars indicate drought (tan) and wet (violet) intervals in the LG-P record. Horizontal bars indicate extreme droughts (red) and wet periods (blue). (a) Dry decades registered in tree ring records from southern Ontario [9]. Asterisks (circles) represent severe (moderate) drought. (b) Percent planktonic diatoms in core LG-P. (c) Megadrought in the West [44]. (d) Periods of extensive droughts and pluvials in the Southwest [38]. Asterisks indicate maximum extent of droughts. (e) "Stine Droughts" and intervening wet period [40]. (f) "Great Drouth" linked to Anasazi Pueblo collapse [38]. (g) Droughts in western North America [34]. (h) Western North American droughts and wet periods [43]. (i) Droughts in the West and Mississippi Valley [44]. (j) Droughts in the Mississippi Valley associated with major cultural disruptions [40].

Tree-ring records from western North America registered widespread, extreme, long-lasting megadroughts during the DWD oscillation following a generally arid period through most of the MCA [4, 38–42]. Aridity in the Adirondacks during the MCA proper was less intense and was not so clearly registered except for an interval of low %P ca. A.D. 950–1000 (Fig 6). Megadroughts occurred during the first dry phase of the DWD interval in much of the Southwest and Midwest, including a "Great Drouth" A.D. 1276–1297 (Fig 6) [38, 43, 44]. The second dry phase was accompanied by megadroughts in the Southwest ca. A.D. 1350–1410 [43], in the West and Mississippi Valley ca. A.D. 1340–1400 [38, 40, 41], and in Mesoamerica ca. A.D. 1380–1400 [45]. An intervening wet period has also been dated to the A.D. 1290–1350 interval in the parts of the West [38, 43], several decades earlier than in the Adirondack records but within the probability ranges of our age models (Fig 6). An opposing wet-dry-wet pattern occurred in the northern Great Plains and Great Lakes regions [21, 46]. Some paleoclimate maps based on such studies also indicate wet conditions in the Northeast during the late 14^th^ century droughts [38, 40, 47] in contrast to the hydroclimate records described here.

### Causal mechanisms

It is likely that both warming (enhanced evaporation) and reduced precipitation contributed to aridity during the dry phases of the DWD oscillation in the Northeast. The oscillation was accompanied by a warm-cool-warm pattern in Maine [15] and in the Northern Hemisphere as a whole during the 14^th^ century [1], and summer precipitation in southeastern Canada decreased during one or both of the warm phases [9, 37]. Reduced soil moisture, water body extent, and evapotranspiration during megadroughts upwind in the west might also have reduced the water vapor content of continental airmasses and thereby contributed somewhat to rainfall reductions downwind in the Northeast. Additional underlying causes of the warm, dry conditions in the Northeast are difficult to identify due to the limitations of available paleoclimate records, but a brief summary of proposed origins of the western megadroughts is offered here in order to illustrate the potential complexity of mechanisms behind the DWD oscillation.

Western megadroughts of the last millennium have been linked to Pacific sea-surface temperatures (SST) through the Pacific Decadal Oscillation (PDO), Pacific North American (PNA) pattern, and El Niño-Southern Oscillation (ENSO) system. Negative phases of the PDO are correlated with drying in the Southwest and parts of the Northeast [42, 44, 48] but apparently not in the Adirondacks (Table 2). Negative excursions occurred ca. A.D. 1300 and A.D. 1400 in one paleo-PDO record [49] but not in others [1, 50].

**Table 2:** Correlations among external forcing mechanisms [56] and monthly weather parameters recorded at two United States Historical Climatology Network stations in the Adirondack uplands from 1950 to 2019 [27].

Parameter	Solar	AO	NAO	PNA	AMO	PDO	SOI
Precipitation							
IL	-0.10 *(0*.*005)*	0.07 *(0*.*02)*	x	x	0.06 *(0*.*04)*	-0.08 *(0*.*01)*	x
DAN	-0.10 *(0*.*003)*	0.08 *(0*.*01)*	x	0.08 *(0*.*01)*	0.11 *(0*.*002)*	x	x
Temperature							
IL	x	0.11 *(0*.*0006)*	0.12 *(0*.*0003)*	0.11 *(0*.*002)*	x	-0.12 *(0*.*0003)*	x
DAN	-0.06 *(0*.*04)*	0.11 *(0*.*0006)*	0.11 *(0*.*0005)*	0.11 *(0*.*0006)*	x	-0.12 *(0*.*0004)*	x

IL, DAN = Indian Lake and Dannemora stations, respectively. P-values in parentheses. x indicates no significant correlation. Solar = solar flux. AO = Arctic Oscillation. NAO = North Atlantic Oscillation. PNA = Pacific North American pattern. AMO = Atlantic Meridional Oscillation. PDO = Pacific Decadal Oscillation. SOI = Southern Oscillation Index.

Positive phases of the PNA pattern are also correlated with drought in the Southwest and Midwest [50] but wetting in the Northeast [12, 16, 48] (Table 2), so continent-spanning aridity during the DWD oscillation seems unlikely to reflect the PNA pattern alone. Paleo-PNA reconstructions are also inconclusive in this regard as one record [12] registered negative values during the first dry phase of the DWD oscillation but not the second, and another [50] yielded only slightly negative values throughout the 13^th^ and 14^th^ centuries.

La Niña-like phases of the ENSO system are associated with aridity in the Southwest and parts of the Northeast [16, 42, 43, 47, 48], but not in the Adirondacks (Table 2). Coral records registered a warm El Niño ca. A.D. 1325–1350 and a strong La Niña ca. A.D. 1390–1400 [51], but paleo-ENSO reconstructions differ from one another [1, 39, 43, 52] so causal links to the DWD oscillation remain speculative.

Atlantic SSTs and solar variability might also have influenced hydroclimates in the Northeast during the DWD oscillation. Warm (positive) phases of the Atlantic Multidecadal Oscillation (AMO) have been linked to the western megadroughts [42, 44, 53] as well as to drying in the Adirondacks (Table 2), and a paleo-AMO reconstruction registered a pronounced warm-cool-warm pattern in the North Atlantic during the 14^th^ century [1]. However, although the Northern Hemisphere’s Arctic Oscillation (AO), which includes the more regional North Atlantic Oscillation (NAO), is positively correlated with temperature and precipitation in much of the Northeast [14, 48] (Table 2), paleo-NAO reconstructions indicate no definitive fluctuations that would explain the DWD oscillation [2, 54]. Periods of reduced solar flux are associated with somewhat drier conditions throughout the Northeast and much of the West [48] (Table 2), so low solar flux during the Wolf Solar Minimum of A.D. 1270–1340 [55] might have contributed to the first dry phase of the DWD oscillation.

In sum, our records help to clarify temporal and geographical aspects of hydroclimate variability in the Northeast during the last millennium, demonstrating at least a temporal linkage to more extreme droughts farther west during the DWD interval. However, the interplay among causal mechanisms behind that variability was probably complex and for now remains uncertain.

### Cultural connections

Regardless of the causes of the DWD oscillation, environmental stresses associated with aridity during the MCA-LIA transition could potentially have impacted indigenous North American cultures in various ways. In western regions, previous analyses have linked severe droughts of the late 13^th^ and 14^th^ centuries to population declines and abandonment of agriculturally dependent settlements in the Four Corners region, the Great Basin, and the Mississippi Valley [38, 44, 50] as well as to Mesoamerican cultural history [42]. In contrast, the hydrological changes inferred for the Northeast during the DWD oscillation as well as during most of the MCA appear to have been milder (Fig 6). That region’s relatively moist climates tend to make lakes and temperate forest habitats less vulnerable to the extreme consequences of hydroclimatic variability faced by western dry-habitat ecosystems, and it is possible that even during periods of reduced precipitation the Northeast could have served as a "hydroclimate refuge" while harsher conditions prevailed elsewhere, particularly if the milder reductions in net water balance also happened to improve local growing conditions for crops that could be harmed by extreme wetness as well as drought.

Archaeological evidence from the Northeast indicates varied and complex changes among diverse cultures during the DWD interval including increased population, sedentism, community building, greater agricultural productivity, and evidence of immigration from afar, some of which could be consistent with the presence of a such a climatic refugium. While the Southwest and Mississippi Valley experienced population declines, central New York and Ontario witnessed the first establishment of centralized longhouse villages with distinctive ceramics and heavy commitment to maize-bean-squash agriculture beginning ca. A.D. 1250–1300 [56–59]. The Uren cultural phase ca. A.D. 1290–1330 (Fig 5) saw eastward migrations into Ontario [60] that, in the context of harsher megadroughts to the west (Fig 6), might also represent indirect effects of differential climatic stress. For example, aridity during the 13^th^ and 14^th^ centuries that contributed to increased warfare and fortifications in the Mississippi Valley [50, 60] could have encouraged emigrations into the Northeast where perennial lakes, forest habitats, and conditions more suitable for agriculture prevailed under less arid climates throughout the last millennium.

Events in single settlements did not necessarily reflect regional cultural stages, but the apparent synchrony of arid phases of the DWD and changes in the cultural history of Crawford Lake, Ontario [36], might be potentially informative in this regard. The first evidence of agriculture near Crawford Lake occurred ca. A.D. 1270 around the time of the first dry phase of the DWD oscillation, and the first large-scale Iroquoian settlement was established ca. A.D. 1325–1375 during the second drought. A later episode of heavy settlement near that lake also co-occurred with another period of reduced %P (i.e. drought) in the record of Little Green Pond ca. A.D. 1410–1450 (Fig 5). However, the possibile existence of causal linkages between the cultural history of Crawford Lake and the relatively mild shifts in hydroclimate revealed in our records remains speculative.

Snow [61] bracketed late Owasco and Oak Hill periods of cultural development in southern Ontario within the A.D. 1250–1400 interval (Fig 5), a time of rapid population growth and increased settlement [60]. Intensification of agriculture and establishment of more permanent year-round villages also occurred among Abenaki cultures of northern Vermont and the Connecticut Valley during the 14^th^ century [62]. The intensification of horticulture and sedentism in the Northeast ca. A.D. 1250 has been thought to reflect climatic cooling [63] but our records suggest that it might also have been influenced by hydroclimate variability during the MCA-LIA transition.

## Conclusions

This study demonstrates the potential for using fine-interval diatom records to help develop a stronger network of hydroclimatic reconstructions in the Northeast, where lacustrine archives are likely to be more readily available than hydroclimatically sensitive tree-ring records. As noted in a recent review of the role of paleoclimatology in modeling future climates [64], many models disagree about the nature and direction of precipitation variability on regional scales as global warming continues. Hydroclimate reconstructions such as these from the Adirondacks can augment shorter instrumental records and help to test and improve such models.

For example, recent concerns that a new megadrought may be emerging in the American Southwest [26] arise in part from reconstructions of past hydroclimate and its presumed forcing mechanisms. If a new continental-scale drought is indeed beginning in this century, then our records suggest that it currently differs from the wider geographic distribution of aridity during the DWD oscillation because climates in the Northeast have generally become wetter, not drier, in recent decades [27, 65].

Our Adirondack records also help to provide a more detailed climatic context for archeological investigations in the region. As a historical analog, the DWD interval suggests that the fluctuations in hydroclimate coincided with significant events in North American cultural history, but their impacts were apparently less severe in the relatively wet Northeast than in the more arid West.
